# Intervention for the Increase in the Uptake of Pneumococcal Vaccination Among Older Patients: A Systematic Review and Meta-Analysis

**DOI:** 10.7759/cureus.82615

**Published:** 2025-04-20

**Authors:** Ryuichi Ohta, Yoshinori Ryu, Chiaki Sano

**Affiliations:** 1 Community Care, Unnan City Hospital, Unnan, JPN; 2 Community Medicine Management, Shimane University Faculty of Medicine, Izumo, JPN

**Keywords:** aged, family medicine, general medicine, health promotion, pneumococcal vaccines, primary healthcare, quality improvement, rural, vaccination coverage

## Abstract

Pneumococcal infection is a significant cause of morbidity and mortality among older adults, with lasting impacts on daily functioning and frailty. Although pneumococcal vaccination is a proven preventive measure, uptake remains low in many primary care settings. This systematic review examined the effectiveness of interventions designed to increase pneumococcal vaccination uptake among older patients in primary care. A comprehensive literature search was conducted in PubMed, Embase, and Web of Science for studies published between January 1995 and April 2025. Studies eligible for inclusion were randomized controlled trials or quasi-experimental studies targeting individuals aged 65 or older. Out of 166 identified records, five studies met the inclusion criteria. Interventions varied widely, including low-literacy educational brochures, computerized physician reminder systems, mailed feedback, point-of-care flyers, and comprehensive system-level programs. All interventions positively affected vaccination uptake, with absolute increases ranging from 1.9% to 35% compared to controls. Strategies targeting healthcare providers, mainly through reminders and feedback mechanisms, tended to yield higher improvements. Only one study was conducted in Asia, indicating a significant gap in regional evidence. The findings suggest that simple and multicomponent interventions can effectively improve pneumococcal vaccination rates in older adults when implemented through primary care. Greater attention to interdisciplinary collaboration and the use of context-specific strategies is warranted. Further trials, particularly in Asian and resource-limited settings, are needed to inform sustainable, scalable approaches to increasing vaccination coverage.

## Introduction and background

Pneumococcal infection is a critical public health concern, particularly among older adults, due to its significant impact on morbidity and mortality [1]. *Streptococcus pneumoniae*, the causative agent, is responsible for various invasive pneumococcal diseases (IPD), such as pneumonia, meningitis, and bacteremia [2]. Older individuals, especially those aged 65 years and above, are at heightened risk due to age-related decline in immune function and the prevalence of underlying comorbidities [3]. According to the European Centre for Disease Prevention and Control (ECDC), the incidence of IPD among adults aged 65 and older was reported to be 18.7 cases per 100,000 population in 2018 [3]. Furthermore, epidemiological data from the United States indicate a 45% increase in IPD incidence among older adults between 2001 and 2007 [4]. Even when treated effectively, pneumococcal infections can lead to long-term declines in functional status among older individuals, contributing to increased frailty and reduced independence [5]. These sequelae underline the importance of prevention strategies to maintain the quality of life in aging populations and highlight the critical need for pneumococcal vaccination.

Pneumococcal vaccination is an effective preventive measure against invasive pneumococcal infections. Studies have demonstrated that the 13-valent pneumococcal conjugate vaccine (PCV13) reduces hospitalizations due to all-cause pneumonia by approximately 10% in older adults [6]. Similarly, the 23-valent pneumococcal polysaccharide vaccine (PPSV23) has decreased significantly pneumonia-related hospitalizations and mortality [7]. Despite these benefits, pneumococcal vaccine coverage remains suboptimal in older populations. For example, a recent study reported that only 26% of older adults who developed IPD had received any pneumococcal vaccination and merely 16% had been vaccinated within the past five years [8]. Barriers to higher uptake include lack of awareness, limited access, misconceptions about vaccine efficacy and safety, and inadequate promotion by healthcare providers [9,10].

Primary care settings are central to delivering preventive healthcare and are pivotal in promoting pneumococcal vaccination. Various strategies have been proposed to enhance vaccination rates in this context, including patient education, reminder systems, proactive provider recommendations, and electronic health record alerts [3,11,12]. While individual studies have evaluated the effectiveness of these interventions, a comprehensive synthesis of the evidence remains limited.

It is essential to systematically assess and compile the existing literature on strategies to improve pneumococcal vaccination uptake in primary care to guide future interventions and policy development. Such evidence is vital for identifying best practices and enhancing implementation at the clinical level. This systematic review and meta-analysis aim to identify and evaluate the effectiveness of interventions conducted in primary care settings that seek to improve pneumococcal vaccination uptake among older adults. The goal is to contribute to higher vaccination rates and improved health outcomes and quality of life in this vulnerable population.

## Review

Methods

Protocol and Registration

This systematic review and meta-analysis followed the Preferred Reporting Items for Systematic Reviews and Meta-Analyses (PRISMA) 2020 guidelines [13]. The review protocol was prospectively registered with the International Prospective Register of Systematic Reviews (PROSPERO) under the registration number CRD420251027192. The protocol includes detailed objectives, inclusion and exclusion criteria, data items, risk of bias assessment methods, and planned statistical analyses.

Eligibility Criteria and Search Strategy

We included randomized controlled trials (RCTs), quasi-experimental studies, and observational studies (e.g., cohort or case-control designs) that assessed the effectiveness of interventions aimed at improving pneumococcal vaccination uptake among older adults (aged 65 years or older) in primary care settings. Studies focusing on general adult populations were included only if data for older adults could be separately extracted. Eligible interventions included, but were not limited to, patient education programs, healthcare provider reminders, electronic health record alerts, financial incentives, and community outreach strategies. Studies not published in English or Japanese, reviews, commentaries, conference abstracts without full text, and those with insufficient data were excluded.

A comprehensive literature search was conducted across the following electronic databases: PubMed/MEDLINE, Embase, and Web of Science. The search strategy included combinations of MeSH terms and free-text keywords such as "pneumococcal vaccination", "older adults", "elderly", "primary care", "intervention", "uptake", and "coverage". The search was limited to studies published between January 1995 and 2025. The concrete search strategies are provided in Table 1.

**Table 1:** Search strategy

Database	Search date	Timeframe	Language restriction	Search strategy	Filters applied
PubMed	2025/4/6	1995/01/01-2025/4/01	English and Japanese	#1 "Pneumococcal Vaccines"[Mesh] OR "pneumococcal vaccine"[tiab] OR "pneumococcal vaccination"[tiab] OR "PCV13"[tiab] OR "PPSV23"[tiab] OR "pneumonia vaccine"[tiab] #2 "Aged"[Mesh] OR "older adults"[tiab] OR "elderly"[tiab] OR "older people"[tiab] OR "geriatric"[tiab] OR "65 years"[tiab] OR "aged 60 and over"[tiab] #3 "Primary Health Care"[Mesh] OR "primary care"[tiab] OR "general practice"[tiab] OR "family medicine"[tiab] OR "clinic"[tiab] #4 "Intervention"[tiab] OR "uptake"[tiab] OR "coverage"[tiab] OR "reminder"[tiab] OR "education"[tiab] OR "strategy"[tiab] OR "promotion"[tiab] OR "program"[tiab] OR "campaign"[tiab] #5 #1 AND #2 AND #3 AND #4	Publication date: 1995/01/01-2025/04/01. Language: English and Japanese. Study types: clinical trials, observational studies, quasi-experimental studies
Embase	2025/4/6	1995/01/01-2025/4/01	English and Japanese	1. 'pneumococcal vaccine'/exp OR 'pneumococcal vaccine':ti,ab OR 'pneumococcal vaccination':ti,ab OR 'pcv13':ti,ab OR 'ppsv23':ti,ab OR 'pneumonia vaccine':ti,ab 2. 'aged'/exp OR 'older adult':ti,ab OR 'elderly':ti,ab OR 'older people':ti,ab OR 'geriatric':ti,ab OR 'aged 60 years':ti,ab OR 'aged 65 years':ti,ab 3. 'primary health care'/exp OR 'primary care':ti,ab OR 'general practice':ti,ab OR 'family medicine':ti,ab OR 'clinic':ti,ab 4. 'intervention study'/exp OR 'intervention':ti,ab OR 'uptake':ti,ab OR 'coverage':ti,ab OR 'reminder':ti,ab OR 'education':ti,ab OR 'strategy':ti,ab OR 'promotion':ti,ab OR 'program':ti,ab OR 'campaign':ti,ab 5. #1 AND #2 AND #3 AND #4 6. [Limit to: Publication Year 1995-2025 and Languages English or Japanese and (Article or Article in Press or Conference Paper)]	Publication year: 1995 to 2025. Language: English and Japanese. Document types: Article, article in press, conference paper
Web of Science Core Collection	2025/4/6	1995/01/01-2025/4/01	English and Japanese	TS=("pneumococcal vaccine" OR "pneumococcal vaccination" OR "PCV13" OR "PPSV23" OR "pneumonia vaccine") AND TS=("older adults" OR "elderly" OR "aged" OR "geriatric" OR "older people" OR "aged 60" OR "aged 65") AND TS=("primary care" OR "general practice" OR "family medicine" OR "clinic" OR "primary health care") AND TS=("intervention" OR "uptake" OR "coverage" OR "reminder" OR "education" OR "strategy" OR "promotion" OR "program" OR "campaign")	Timespan: 1995-2025. Languages: English and Japanese. Document types: articles, proceeding papers

Data Extraction and Management

Two independent reviewers (RO and YR) screened titles and abstracts, followed by full-text screening of potentially eligible studies. Disagreements were resolved through discussion or consultation with a third reviewer. A standardized data extraction form was used to collect study characteristics (e.g., author, year, country, setting), population details (e.g., age, comorbidities), intervention features, control conditions, outcomes related to pneumococcal vaccine uptake, and follow-up duration. Data were entered into Excel and cross-checked for consistency.

Risk of Bias Assessment

Two reviewers independently assessed the risk of bias for included studies using the Cochrane Risk of Bias tool (RoB 2.0) for RCTs and the Risk of Bias in Non-randomized Studies of Interventions (ROBINS-I) tool for non-randomized studies [14]. Any disagreements were discussed and resolved through consensus. The overall risk of bias was categorized as low, some concerns, or high, and findings were tabulated.

Outcomes and Data Synthesis

The primary outcome was the proportion of older adults who received pneumococcal vaccination after implementation of the intervention, compared to the control or baseline period. Where possible, risk ratios (RRs) or odds ratios (ORs) and their 95% confidence intervals (CIs) were extracted or calculated. Due to anticipated heterogeneity among interventions and settings, a random-effects meta-analysis was conducted using the DerSimonian and Laird method. Heterogeneity was assessed using the I² statistic, with values above 50% indicating substantial heterogeneity. Where quantitative synthesis was not feasible, a narrative synthesis was provided.

Results

Study Selection

**Figure 1:**
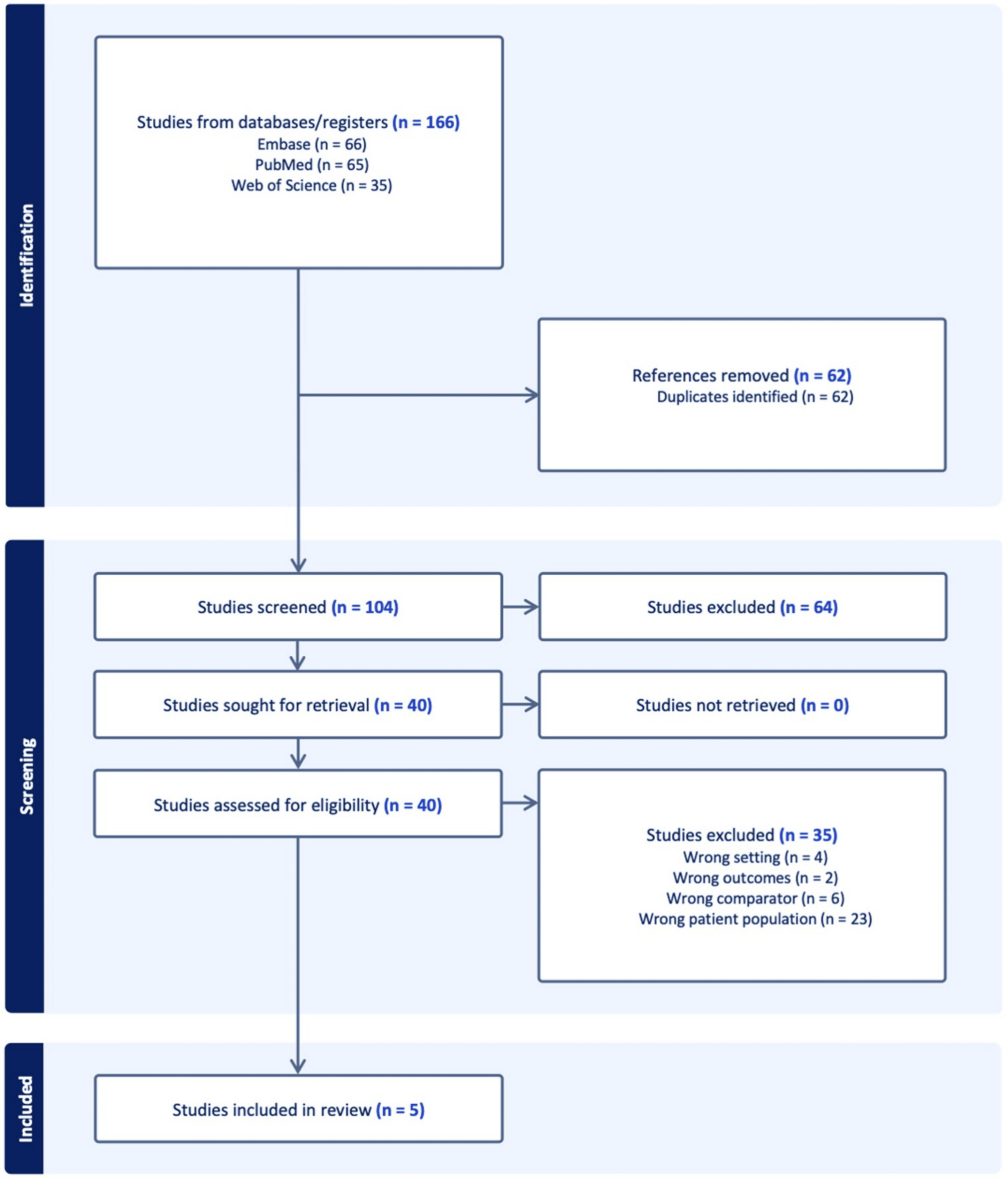
Study selection flow

A total of 166 records were identified through database searches, including 66 from Embase, 65 from PubMed, and 35 from Web of Science. No additional records were retrieved through citation searching or grey literature sources. After 62 duplicates were removed via Covidence, 104 records remained for title and abstract screening. Of these, 64 studies were excluded, leaving 40 full-text articles assessed for eligibility. Following full-text review, 35 studies were excluded for the following reasons: inappropriate patient population (n=23), wrong comparator (n=6), wrong setting (n=4), and irrelevant outcomes (n=2). Five studies met the inclusion criteria and were included in the final systematic review. No ongoing studies or studies awaiting classification were identified (Figure 1).

Study Characteristics

This review included five studies published between 1999 and 2019. All studies were RCTs or cluster RCTs assessing the effectiveness of interventions aimed at increasing pneumococcal vaccination uptake among older adults (aged ≥65 years). The studies were conducted in diverse settings, including the United States (four studies), Hong Kong (one study), and Singapore (one study).

The characteristics of the included studies are summarized in Table 2. Sample sizes varied widely, ranging from 433 individuals in a single outpatient clinic to over 18,000 older adults across 25 primary care clinics. Interventions included patient-directed educational tools (e.g., low-literacy brochures, flyers), healthcare provider-targeted strategies (e.g., computerized reminders), and system-wide quality improvement programs (e.g., the 4 Pillars Practice Transformation Program). Control groups typically received standard care, such as usual clinical workflows, non-targeted health education materials, or no intervention.

**Table 2:** Included study characteristics USA: United States of America; RCT: randomized controlled trial; PPV: pneumococcal polysaccharide vaccine; QI: quality improvement; GP: general practice

Author (year)	Country	Study design	Participant description	Intervention description	Intervention (events/N, %)	Control (events/N, %)
Jacobson et al. (1999) [15]	USA	RCT	433 older adults (65 years old or over or with chronic diseases) in a public primary care clinic	1-page low-literacy educational brochure encouraging PPV	44/221 (19.9%)	8/212 (3.8%)
Dexter et al. (2001) [16]	USA	RCT	6,371 hospitalized patients (65 years old or over and/or PPV-eligible) in an urban public hospital	Computerized physician reminder system during order entry	607/1,696 (35.8%)	18/2,314 (0.8%)
Chan et al. (2015) [17]	USA	Cluster RCT	2,517 older outpatients (65 years old or over) with chronic diseases attending five public clinics	Nurse-delivered 3-minute telephone+3-minute face-to-face education vs. standard care (leaflets/video)	716/1,251 (57.2%)	609/1,266 (48.1%)
Zimmerman et al. (2017) [18]	USA	Cluster RCT	13,314 older patients (65 years old or over) seen in 25 primary care practices	4 Pillars Transformation Program (system-level multicomponent QI strategy)	5,729/7,219 (79.3%)	4,519/6,095 (74.1%)
Ho et al. (2019) [19]	Singapore	Cluster RCT	8,837 patients 65 years old or over visiting 22 GP clinics	Point-of-care educational flyer at the clinic registration desk	249/4,378 (5.7%)	165/4,459 (3.7%)

All studies reported vaccination outcomes using individual or cluster-level vaccination uptake data, with intervention effects reported as ORs or derived from raw event counts. Most interventions were implemented in outpatient settings, except for Dexter et al. [16], which targeted hospitalized patients during inpatient stays. Notably, Chan et al. [17] included telephone and face-to-face educational components delivered by nurses in public outpatient clinics in Hong Kong (Table 2).

Risk of Bias Within Studies

**Figure 2:**
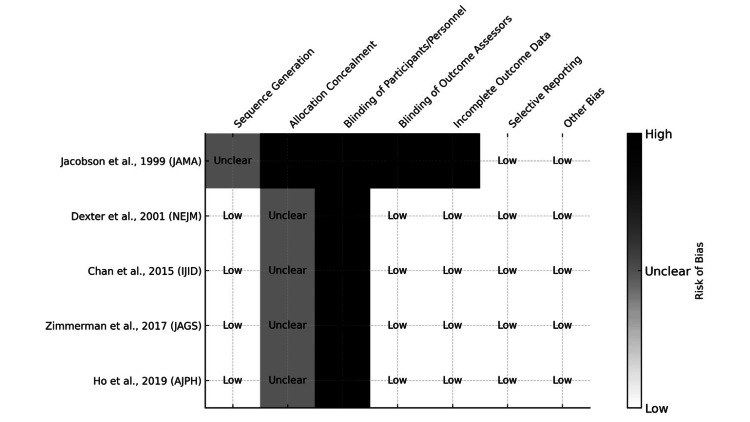
Risk of bias assessment of included randomized controlled trials The assessment was conducted using the Cochrane Risk of Bias Tool (RoB 2.0) for five included studies [15-19]. Most studies demonstrated low risk in outcome reporting and data completeness, while performance bias was high due to the non-blinded nature of behavioral interventions. References [15-19] correspond to Jacobson et al. (1999), Dexter et al. (2001), Chan et al. (2015), Zimmerman et al. (2017), and Ho et al. (2019), respectively. JAMA: Journal of the American Medical Association; NEJM: New England Journal of Medicine; IJID: International Journal of Infectious Diseases; JAGS: Journal of the American Geriatrics Society; AJPH: American Journal of Public Health

The risk of bias was assessed across seven standard domains for all included RCTs using the Cochrane Risk of Bias Tool. As shown in Figure 2, all studies appropriately addressed outcome reporting and handling of incomplete data, resulting in low risk for these domains. However, blinding participants and personnel was a consistent challenge across all five trials due to the nature of behavioral and educational interventions, leading to a high risk of performance bias. Four of the five studies clearly described sequence generation methods, but allocation concealment procedures were often inadequately reported. Despite these limitations, all studies used objective outcome data (e.g., EMRs), which reduced detection bias. Overall, while performance and allocation biases were of concern in some studies, the risk of bias was generally low to moderate, supporting the validity of the results (Figure 2).

Results of Individual Studies and Synthesis

This systematic review included five RCTs and cluster RCTs evaluating interventions to improve pneumococcal vaccination uptake among older adults (aged ≥65 years). The studies were conducted in the United States, Hong Kong, and Singapore between 1999 and 2019 and involved a variety of settings, including inpatient, outpatient, and primary care environments.

Intervention strategies varied in their target and complexity. Jacobson et al. [15] evaluated a low-literacy educational brochure and reported an OR of 5.28 (95% CI: 2.80-9.93). Dexter et al. [16] demonstrated a marked improvement using a computerized reminder system for inpatient physicians, with an OR of 68.37 (95% CI: 42.69-109.51). Chan et al. [17] assessed a nurse-delivered brief education program and observed a moderate effect (OR: 1.20; 95% CI: 1.06-1.37). Zimmerman et al. [18] evaluated a multicomponent quality improvement program (4 Pillars Practice Transformation) and reported an OR of 1.29 (95% CI: 1.21-1.37). Finally, Ho et al. [19] found that point-of-care flyers at clinic registration increased vaccine uptake modestly (OR: 1.78; 95% CI: 1.39-2.29).

**Figure 3:**
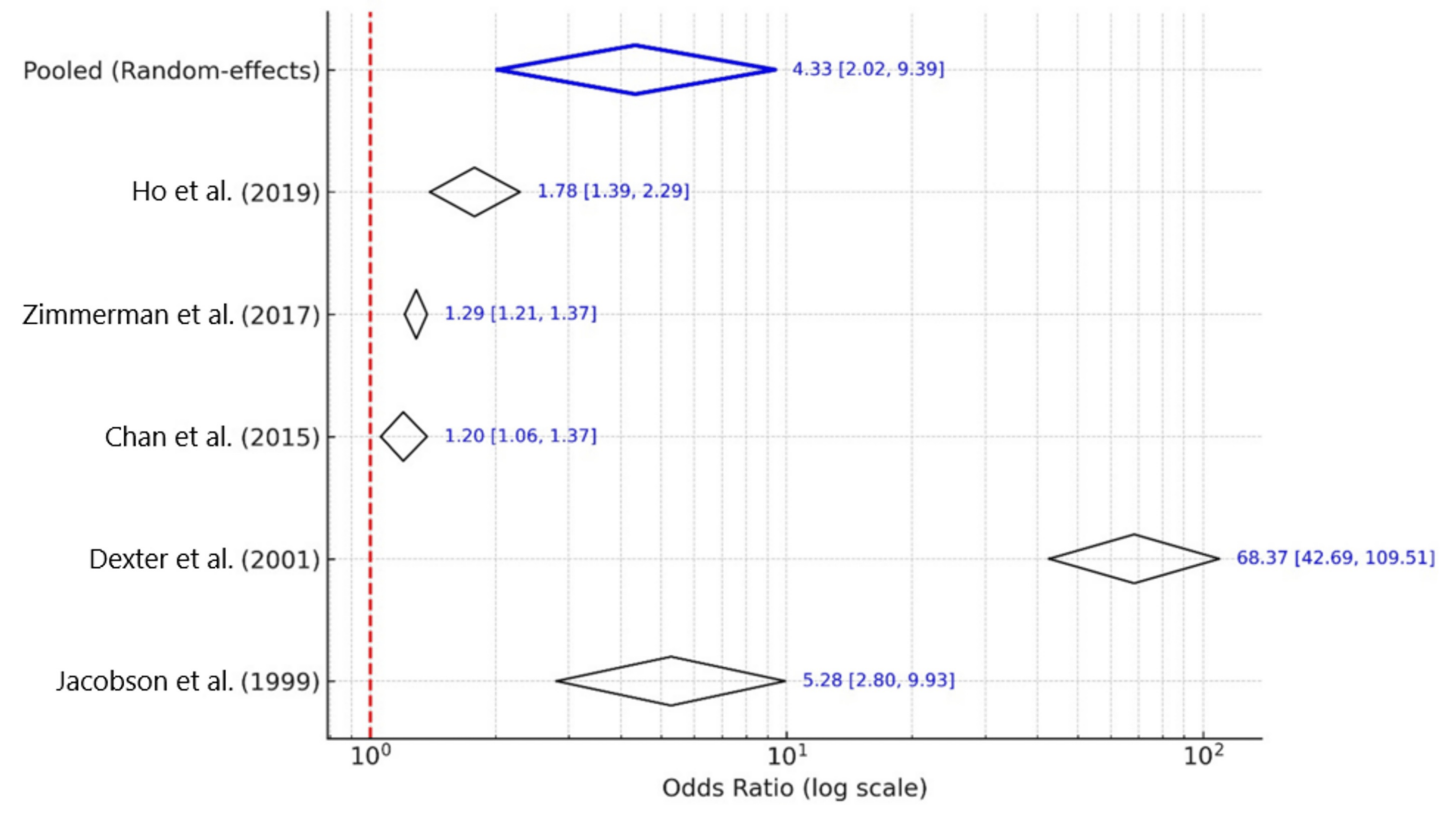
Forest plot with diamonds for individual studies and pooled estimate The five included studies [15-19] each demonstrated a positive effect, with a pooled OR calculated using a random-effects model. OR: odds ratio

A random-effects meta-analysis yielded a pooled OR of 4.33 (95% CI: 2.02-9.39), suggesting that interventions significantly improved pneumococcal vaccination rates among older adults. However, substantial heterogeneity was observed (I²=98.5%), likely due to differences in intervention design, populations, and healthcare settings. All studies showed directionally positive effects favoring the intervention group, as illustrated in the forest plot (Figure 3).

Discussion

Summary of Main Findings

This systematic review and meta-analysis synthesized evidence from five RCTs and cluster RCTs conducted in the United States, Hong Kong, and Singapore to evaluate the interventions' effectiveness in increasing pneumococcal vaccination uptake among older adults in primary care and outpatient settings. Despite considerable variability in design and delivery, all included studies reported a statistically significant improvement in vaccine uptake in the intervention arms compared to controls [17-19]. The pooled analysis demonstrated that interventions were associated with a more than fourfold increase in vaccination likelihood (OR: 4.33; 95% CI: 2.02-9.39), although heterogeneity was substantial (I²=98.5%). Interventions ranged from low-cost, easily implementable strategies, such as low-literacy educational brochures and point-of-care flyers, to more resource-intensive approaches, like computerized physician reminders and multicomponent practice transformation programs [15,16]. Even brief, nurse-delivered education sessions led to meaningful improvements, highlighting the potential of human-centered communication strategies.

Comparison with Previous Literature

Our findings align with prior systematic reviews and immunization guidelines that emphasize the utility of provider prompts, reminder systems, and standing orders to enhance adult vaccination rates [20,21]. However, this study contributes uniquely by focusing specifically on pneumococcal vaccination among older adults and including data from both Western and Asian healthcare systems. The inclusion of studies from Hong Kong and Singapore provides important cross-regional insights. However, the dominance of US-based studies underscores a continuing research gap in low- and middle-income countries (LMICs), particularly in Asia, where pneumococcal disease burden is high and vaccine coverage remains suboptimal [22,23].

Moreover, the results support the growing consensus that multicomponent, system-level interventions, such as the 4 Pillars Practice Transformation Program, are more effective and sustainable than single-point efforts. Integration into electronic health records and clinical workflows appears to be a key facilitator of success [24,25].

Clinical and Academic Implications

This review underscores the central role of primary care systems in improving adult immunization through scalable, evidence-based interventions. Brief and targeted education tools, provider alerts, and nurse-led counseling can be readily incorporated into existing workflows without significant resource demands [26]. Interventions involving interprofessional collaboration, engaging physicians, nurses, medical assistants, and clerical staff, may be especially beneficial in reaching high-risk or underserved populations [27,28].

Academically, this synthesis highlights the need for continued research in implementation science, especially in non-Western contexts. Evaluations should focus on interventions' cultural adaptability, sustainability, and cost-effectiveness. Furthermore, quality improvement frameworks should be explored to scale effective practices across healthcare systems and regions [29].

Limitations

This review has several limitations. First, the number of eligible studies was limited (n=5), and most were conducted in high-income countries, potentially limiting generalizability to LMICs. Second, despite directionally consistent results, considerable clinical and methodological heterogeneity among the interventions, settings, and populations likely contributed to the high I² value. Third, differences in outcome definitions, vaccination eligibility criteria, and adjustment for confounding factors (e.g., comorbidities, socioeconomic status) may affect comparability. Lastly, publication bias remains a concern, as studies with negative or null results may be underreported.

Future Directions

To address the research gap in LMICs and culturally diverse populations, future studies should prioritize randomized trials and quality improvement initiatives in Asian and other underrepresented regions. Particular attention should be paid to interventions that leverage digital health platforms, task-sharing with nurses or community health workers, and culturally adapted messaging. Long-term follow-up and cost-effectiveness analyses will inform health policy and funding allocation.

## Conclusions

This review demonstrates that diverse interventions, including simple educational materials, provider prompts, and system-wide transformation strategies, effectively increase pneumococcal vaccination uptake among older adults in primary care settings. While context and intensity vary, all reviewed interventions led to measurable improvements. The findings support integrating reminder systems and interprofessional strategies into routine care. However, the lack of data from low-resource and non-Western settings highlights a critical need for global research efforts to promote equitable vaccine delivery and protection of vulnerable populations.
